# Post-traumatic stress disorder in adolescents during the Covid-19 pandemic: a cross-sectional study of 326 cases

**DOI:** 10.1192/j.eurpsy.2024.1045

**Published:** 2024-08-27

**Authors:** K. Mayssa, B. Jaweher, K. Ali, B. T. Donia, H. Imen, A. Hela, K. Khaoula, M. Yousr

**Affiliations:** Department of Child Psychiatry, University Hospital of Hedi Chaker, Sfax, Tunisia

## Abstract

**Introduction:**

Since December 2019, the coronavirus pandemic has led to the deaths of almost 4.37 million people worldwide and 21,905 people in Tunisia. Containment measures, stress due to fear of infection by the virus and death are likely to be traumatic events, particularly in adolescents, and may lead to the development of symptoms of post-traumatic stress disorder (PTSD).

**Objectives:**

To determine the prevalence of PTSD in a population of adolescents during the COVID-19 pandemic and to identify the factors associated with it.

**Methods:**

This study was a cross-sectional among a representative sample of students enrolled in secondary schools, in the region of Hamma- Gabes. We used a pre-established information sheet comprising 27 questions exploring sociodemographic and family data and specific data relating to the COVID-19 pandemic. The Arabic version of The Child PTSD Symptom Scale (CPSS) was used to screen for PTSD symptoms.

**Results:**

326 adolescents were collected which the mean age was 16.6 years (14 to 18 years). The family environment was conflictual in 11.9% of cases. Among the adolescents, 5.5% had a history of somatic pathology. A history of psychiatric pathology was noted in 0.6%, dominated by depression. Personal infection by Covid-19 was noted in 4% of adolescents. A family member was affected in 27.3% of cases. Adolescents were exposed to the death of a close relative in 22.4% of cases. PTSD was diagnosed (according to the CPSS) in 37.4% of cases, with mild severity in 6.5%, moderate in 0.6%, moderately severe in 8%, severe in 5.2% and extremely severe in 17.2%. The analytical study showed that PTSD was correlated with a conflictual family environment (p=0.017), personal infection by COVID (P=0.003), infection of a close relative by COVID (P<0.001) and the death of a close relative by COVID (p<0.001).

**Conclusions:**

According to our study, the frequency of post-traumatic stress disorder among adolescents during the COVID-19 pandemic was high, underlining the need to screen at-risk populations for populations for early intervention.

**Disclosure of Interest:**

None Declared

